# Metabolic trade-offs between biomass synthesis and photosynthate export at different light intensities in a genome–scale metabolic model of rice

**DOI:** 10.3389/fpls.2014.00656

**Published:** 2014-11-28

**Authors:** Mark G. Poolman, Sudip Kundu, Rahul Shaw, David A. Fell

**Affiliations:** ^1^Cell Systems Modelling Group, Department of Biological and Medical Science, Oxford Brookes UniversityOxford, UK; ^2^Department of Biophysics, Molecular Biology, and Bioinformatics, Calcutta UniversityKolkata, India

**Keywords:** rice, flux balance analysis, metabolic modeling, photosynthesis, mitochondrial metabolism

## Abstract

Previously we have used a genome scale model of rice metabolism to describe how metabolism reconfigures at different light intensities in an expanding leaf of rice. Although this established that the metabolism of the leaf was adequately represented, in the model, the scenario was not that of the typical function of the leaf—to provide material for the rest of the plant. Here we extend our analysis to explore the transition to a source leaf as export of photosynthate increases at the expense of making leaf biomass precursors, again as a function of light intensity. In particular we investigate whether, when the leaf is making a smaller range of compounds for export to the phloem, the same changes occur in the interactions between mitochondrial and chloroplast metabolism as seen in biomass synthesis for growth when light intensity increases. Our results show that the same changes occur qualitatively, though there are slight quantitative differences reflecting differences in the energy and redox requirements for the different metabolic outputs.

## 1. Introduction

Genome–scale metabolic modeling (GSM) is a technique for investigating the feasible metabolic states of an organism, taking account of the possibilities and constraints imposed by the structure of the metabolic network. The principal constraint is that of mass balance, dictated by the stoichiometries of the reactions in the network, coupled with the assumption of a metabolic steady state where the production and consumption of all internal metabolites of the network is balanced so that their concentrations remain constant. This defines a space containing all feasible states of the metabolism, from which more specific solutions are extracted with a combination of additional constraints, such as experimentally-observed values of nutrient uptake and growth rates, with an objective function that is intended to define an optimal state of the metabolism. The most common objective function, particularly for the models of micro-organisms that constitute the majority of genome–scale models, is optimization of the growth yield (Schuster et al., 2008). Another candidate for the objective function is minimization of total flux in the network (Holzhütter, 2004; Poolman et al., 2009), acting as a proxy for economy in the enzymatic machinery. The models are solved using linear programming, and associated techniques that are part of the methodology known as Flux Balance Analysis (FBA), to give a predicted distribution of fluxes in the metabolic network.

The majority of GSM has been applied to microorganisms, including photosynthetic organisms such as cyanobacteria and *Chlamydomonas* (Boyle and Morgan, 2009; Chang et al., 2011). Our initial model of *Arabidopsis thaliana* metabolism (Poolman et al., 2009) was applied to growing cells of a root tissue culture, which can be regarded as essentially similar to a microbial culture and analyzed for efficient growth. By comparing the predicted fluxes with fluxes measured on the same cells by ^13^C metabolic flux analysis, we showed (Cheung et al., 2013) that minimization of total flux gave the best flux predictions and also that cell maintenance involved a significant requirement for NADPH as well as the ATP that is assumed to be the main maintenance requirement for microorganisms. Generally, however, the modeling of multicellular higher plants by FBA requires more subtle and diverse objectives.

Coupled to this consideration is the metabolic specialization of the tissues. Mintz-Oron et al. (2012) created tissue-specific variants of their Arabidopsis model by linking their genome–scale reconstruction to protein and transcript levels. They used the seed variant to model experimental flux data on developing embryos—again, a growing tissue. For a fully-expanded mature leaf, the metabolic objective has to involve the provision of photosynthate to the rest of the plant. In addition, this objective must be met over the diurnal cycle of light and dark. Cheung et al. (2014) described a method for finding the optimum metabolism for a leaf to provide nutrients for the phloem both day and night, with the dark metabolism using metabolites (not just starch) stored during the light. de Oliveira Dal'Molin et al. (2010) derived mesophyll and bundle sheath cell specific models from their genome–scale model of C_4_ plant metabolism, C4GEM, and modeled their interactions as they produced biomass, starch, and sucrose for export in the light. Their model autonomously reproduced the classical C_4_ pathway and its effects on organelle function in the two cell types. Grafahrend-Belau et al. (2009) built a central carbon metabolism model of barley endosperm (though their model was hand-constructed rather than based on an annotated genome) and used FBA to model grain yield under different circumstances. Their predictions of growth rates and metabolic patterns from modeling anoxic, hypoxic, and aerobic conditions are consistent with experimental observations.

Our GSM of rice (*Oryza sativa indica*) (Poolman et al., 2013) represented a mesophyll cell of an expanding leaf that had to photosynthesis to produce biomass components for growth, as well as intracellular starch and sucrose, across a range of light intensities, but it was not assumed to be exporting metabolites to the phloem to supply the rest of the plant. Our results showed that there were several rearrangements of metabolic patterns involving changes in the interactions between mitochondrial and chloroplast metabolism as light intensity increases, with mitochondria supplementing ATP production at low light intensities. The latter observation in particular is consistent with the experimental evidence reviewed by Padmasree et al. (2002), though the experiments are typically performed on mature source leaves. Another factor in the metabolic transitions was whether NH_3_ or NO_3_ was used as the N source, because the latter has a higher energy requirement, but again the experimental consequences have typically been studied on mature leaves (e.g., Searles and Bloom, 2003). Since the photosynthetic metabolism of a source leaf is focused on making a smaller range of compounds for export to the phloem than are needed for production of leaf biomass, we needed to determine whether equivalent transitions in metabolic patterns also occur when the model is altered to represent a cell producing phloem sap. Apart from being a more physiologically relevant scenario, this is also a necessary step on the way to making a whole plant metabolic model. As cessation of growth and starting to export photosynthate is unlikely to be an instantaneous step change, we also modeled a progressive transition between the two states across the range of light intensities to see whether this occurred smoothly or whether there were thresholds where the flux pattern change abruptly.

## 2. Methods

The model of rice metabolism of a rice mesophyll cell used in this study is that we reported previously (Poolman et al., 2013) and is available in the supplementary data to that paper (http://dx.doi.org/10.1104/pp.113.216762). In brief, it was based on the genome annotation recorded in the“RiceCyc” database, version 2.01, downloaded from http://www.gramene.org/pathway/ricecyc.html (Youens-Clark et al., 2011) derived from International Rice Genome Sequencing Project (2005)'s annotation of the *Oryza sativa japonica* cv. Nipponbare genome sequence. This resulted in a model with 1484 metabolites in 1736 reactions, of which 1029 could carry flux from nutrients to the specified biomass components, and at least 790 reactions had gene associations.

All computation was achieved using the software package ScrumPy–metabolic modeling in Python Poolman (2006). This includes facilities for performing linear programming using the Gnu Linear Programming Kit– (http://www.gnu.org/software/glpk/) and interrogating BioCyc flat-file databases. The package, and further information, can be obtained from http://mudshark.brookes.ac.uk/ScrumPy or by contacting MGP.

The inputs to the model metabolic network are photons, water, CO_2_, nitrogen as NH_3_ and NO_3_, phosphate and sulfate, implemented as transport reactions bringing the species into the cell. Similarly, the outputs of metabolism are also formally expressed as transporters to an external sink, even for cellular constituents such as biomass, starch, and vacuolar sucrose that are in reality intracellular. In contrast to common practice in flux balance modeling, we do not include within the model a reaction equation that takes all the monomers needed for biomass as reactants in amounts proportional to their occurrence in measured biomass. This is in order to maintain the distinction between the structure of the network model and the experimental data and constraints that are applied in its analysis, which is especially relevant in this case where we want to change the metabolic output to something other than biomass. Other issues with the standard biomass equation formulation, causing imprecision in the FBA solutions, have recently been identified (Chindelevitch et al., 2014). Instead of a biomass equation, we use the measured composition of biomass plus growth rate, and in this case phloem sap as well, to compute the relative rates of export of the constituent components of biomass and phloem contents, and apply these as constraints to the linear programming problem.

The composition of leaf biomass was taken as previously from data by Juliano (1986), except for the nucleic acid content and composition, which were estimated from the results of Kwon and Soh (1985). The composition of rice phloem sap was taken from Hayashi and Chino (1990). In order to make a controlled comparison between metabolism producing biomass and that producing phloem sap, we set the rate of phloem production to require net fixation of the same amount of CO_2_ as biomass. The transporter fluxes for the two outputs are listed in Supplementary Tables 1, 2. Note that though a leaf cell may vary the composition of its output to the phloem, and its quantity relative to chloroplastic starch, according to light intensity, we are not attempting to make these variables of the modeling performed here.

As previously, we have analyzed the responses of the optimal, minimal flux solution of the linear program to variation in light intensity. The range investigated was from zero (where, obviously no solution is found) up to the point beyond which all flux responses remain linear. The minimum flux below which no solution is possible was identified from a simple bisection search. However, in this case we also carried out a second scan whereby the proportion of biomass in the output went from 100% to zero, whilst the phloem output went from 0 to 100%. The 100% phloem output rate was set to contain the same amount of fixed carbon as the maximum biomass output, so that the amount of carbon fixed did not change along this second scan axis. The reason for carrying out a 2-D scan of light intensity and fraction of metabolic output to the phloem was two-fold. Firstly, it seems likely that output to the phloem will increase gradually as growth slows, so that a mixed output will exist for a certain period during maturation of a mesophyll cell. Secondly, given that scans of a single parameter give discontinuities in the set of reactions involved in the solution (c.f. our previous study, Poolman et al., 2013), there exists the possibility that this could also occur along the transition from biomass to phloem output.

Two additional constraints were imposed, as previously, on the solutions. The first is that the cyclic photophosphorylation is not allowed to exceed the non-cyclic rate. This is to ensure that the model displays a response to over-reduction at high light intensities, since otherwise the solutions would escape this by passing all excess photons to cyclic photophosphorylation, and trivially counteracting the excess ATP production by futile cycling (such as starch synthesis and degradation in the chloroplast). The second is an arbitrary limit set on the sum of the rubisco carboxylase and oxygenase reaction rates, to implement a limit on the Calvin cycle flux. This is not based on a presumption that rubisco alone implements an upper limit to the Calvin cycle, only that such a limit exists; as this is not a kinetic model, the limitation could be equally applied to any of the enzymes in the same enzyme subset as rubisco to the same effect. We also assumed a fixed ATP requirement for growth-associated and non-growth associated maintenance. In all these cases, the value associated with the constraint affects the photon flux at which transitions in metabolism occur, but does not change the existence or the nature of the transition.

To calculate plots of relative flux differences, *D*, any reaction flux *J*_*i*,*r*_ measured at light intensity *i* and fraction of phloem output *r* was expressed as a relative difference to the same flux at the same light intensity for *r* = 0.5, i.e.,:

(1) $$D_{i,r}=\frac{J_{i,r}}{J_{i,r=0.5}}-1$$

## 3. Results

**Figure 1:**
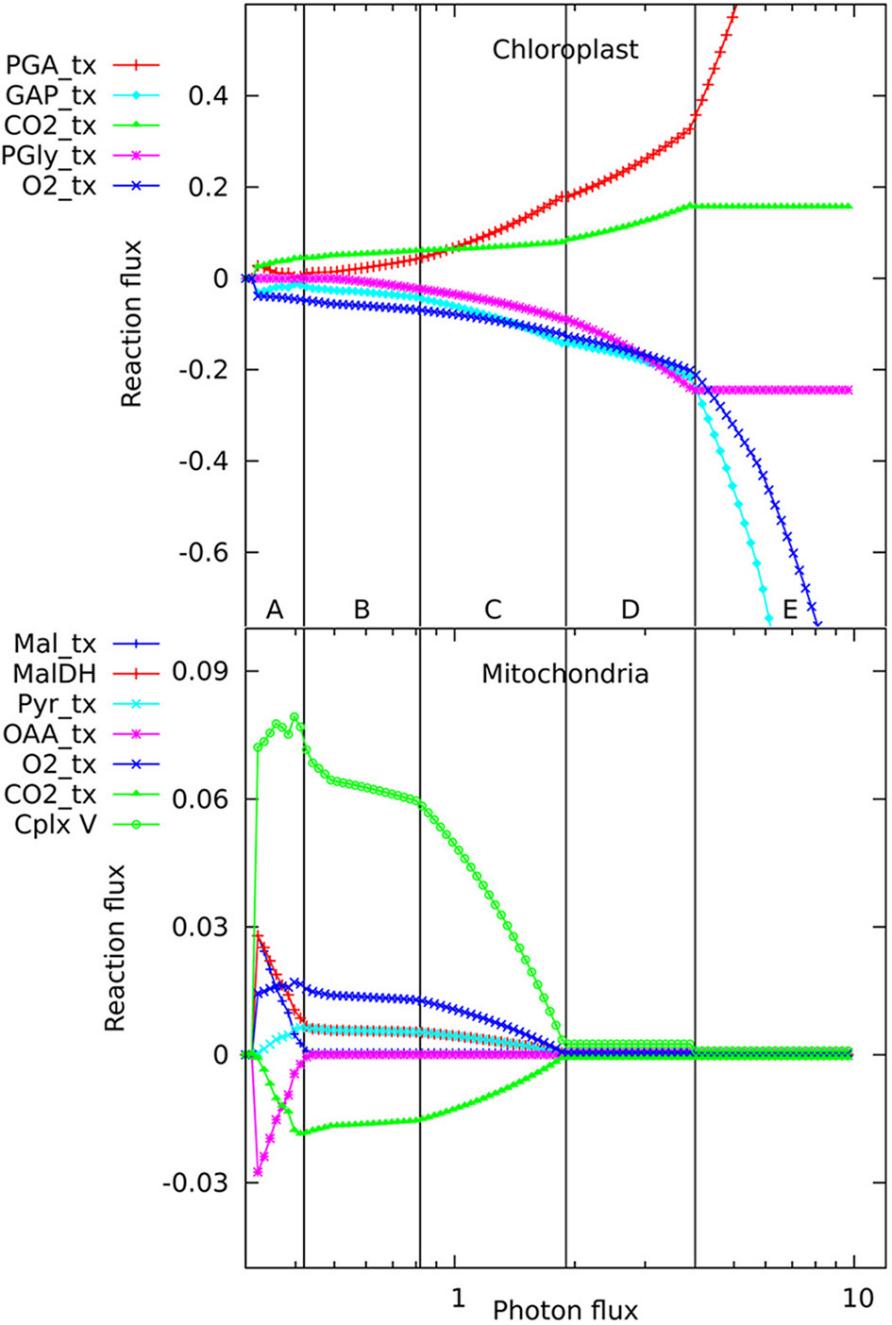
**Responses of solutions in the chloroplast and mitochondrion to varying photon fluxes**. Suffix _tx indicates transport of the named metabolite, positive values represent import to the compartment, negative values export. The abscissa is plotted as a logarithmic scale to enable the full set of responses to be easily seen; this causes the reaction responses to appear as curves, whereas they vary linearly with light intensity, showing abrupt changes in slope where the pattern of fluxes changes. The four major flux rearrangements are indicated by vertical lines, dividing the flux patterns into five major regions labeled A–E.

The number of reactions active in the metabolic network when producing phloem sap is between 173 and 180, depending on light intensity, which is, not surprisingly, lower than the 270–277 needed to make the major biomass components. When varying the light intensity for a mature, non-growing source leaf, we observed five distinct regions in the flux responses in mitochondria and chloroplasts (Figure 1) that appeared qualitatively similar to the pattern of responses we reported previously for a growing, non-exporting leaf (Poolman et al., 2013). At these transitions there are changes in the set of reactions constituting the optimal solution for the flux distribution. Starting from the lowest light level sufficient to produce the phloem sap, in regions A and B, ATP is generated by the mitochondria (shown by the flux in complex V, the ATP synthase), with this flux falling away in region C. The ATP synthesis is driven by carbon and reductant exported from the chloroplast; hence, there are corresponding changes in the chloroplast transport reactions. A corollary is that some of the photosynthetic O_2_ is utilized by mitochondrial respiration, whilst the CO_2_ evolved by respiration is refixed in the chloroplast. In region A, most mitochondrial flux is accounted for by generation of ATP through oxidation of NADH supplied primarily from the operation of a mitochondrial malate-oxaloacetate shunt driven by export for reductant from the chloroplast. Region B is characterized by complete oxidation of pyruvate by the conventional operation of the citric acid cycle and the mitochondrial electron transport chain with increased CO_2_ fixation in the chloroplast as the respiratory products are recycled (Poolman et al., 2013). Region C is the transition between B and D. In region D, ATP production and export by the chloroplast has increased and largely replaces mitochondrial production. Photorespiration is also increasing throughout region D, along with increased recycling of CO_2_ until the Calvin cycle saturates, triggering the transition to region E where excess reductant and ATP are dissipated by substrate cycles. In fact, the transitions between regions A, B, and C (Figure 1) occur at the same light intensities as when biomass is being produced, and the differences in energy and redox input needed to make the phloem contents rather than leaf biomass only make small changes to the positions of transitions between regions C, D, and E. Hence it seems that the transitions are an intrinsic feature of how the central core of metabolism rearranges to balance energy and redox requirements at different levels of light input, and the specific destination of the fixed carbon has only a minor influence.

**Figure 2:**
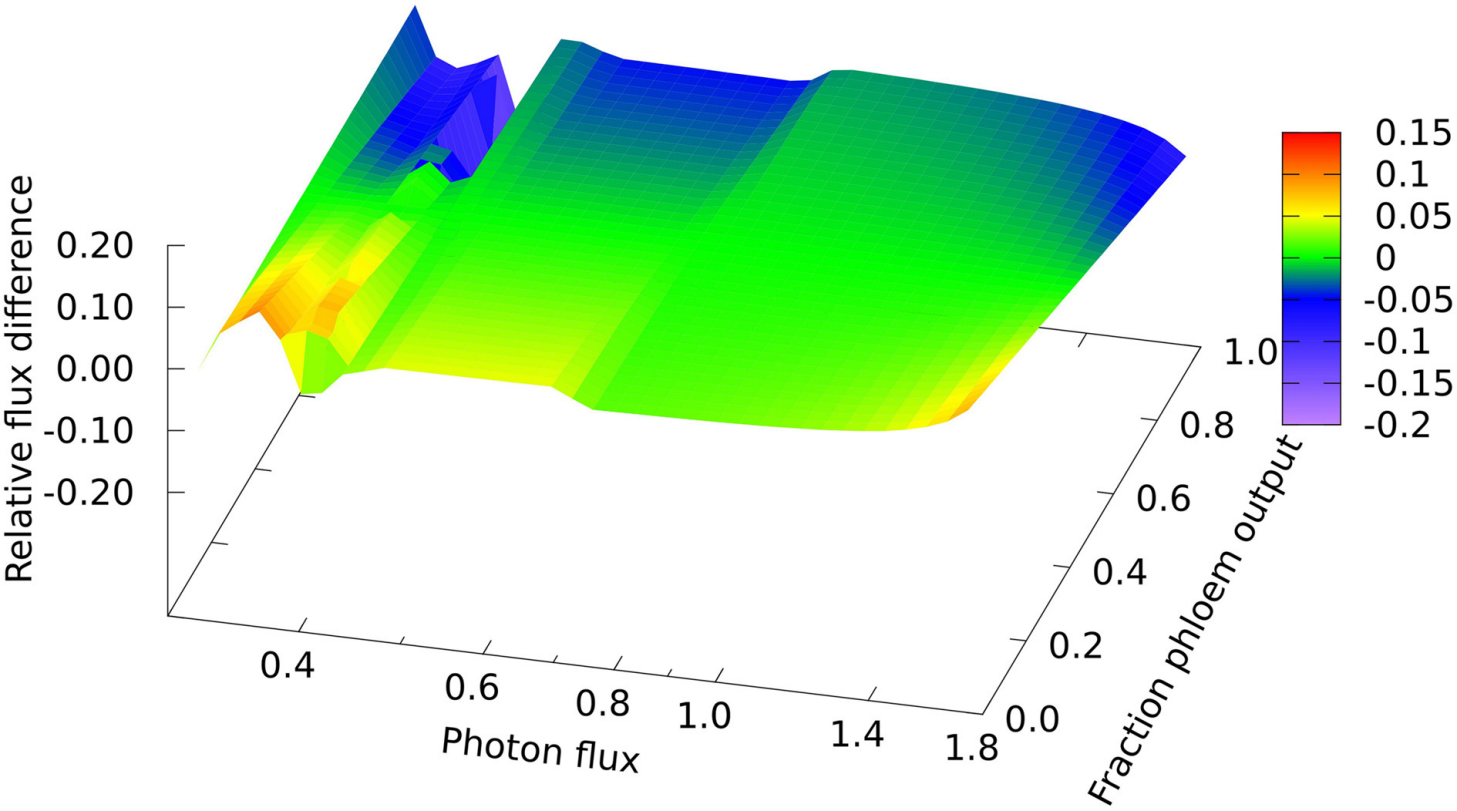
**Responses of mitochondrial malate dehydrogenase flux to leaf output and light intensity**. The plot is of the relative flux difference compared to the flux for production of equal amounts of biomass and phloem sap as given in Equation (1) in the Methods section. The photon flux axis is scaled logarithmically and covers regions A, B, and C. The y–axis represents a linear transition of the leaf's metabolic output from being wholly used for growth to being wholly used for production of phloem sap.

**Figure 3:**
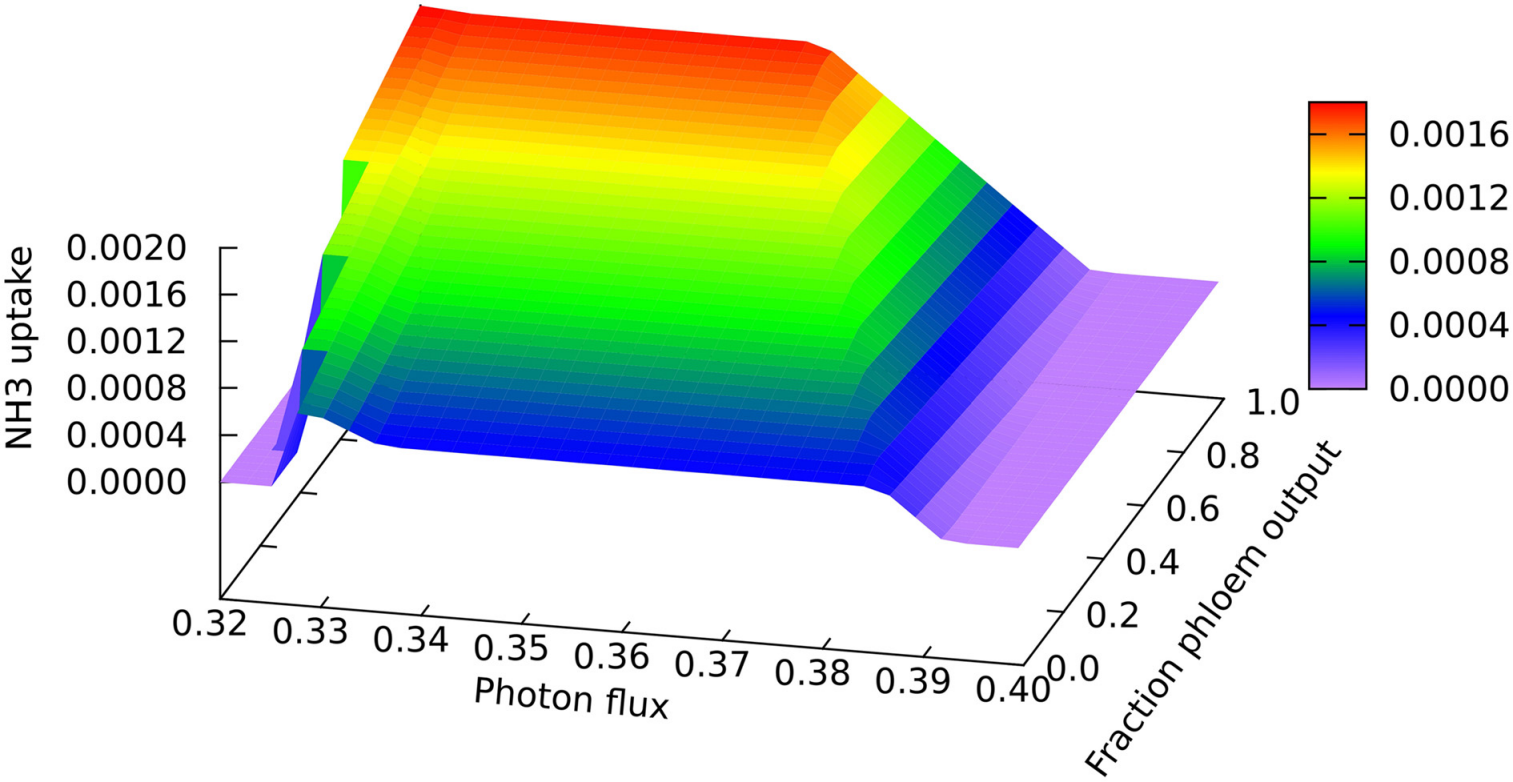
**Responses of the ammonia uptake flux to leaf output and light intensity**. The photon flux axis is light intensity from its minimal necessary value where only NH_3_ is used as N-source to a value just above the transition to full NO_3_ utilization in region A, represented by the zero NH_3_ flux on the right of the graph. The zero flux area in the bottom left corner is where there is no feasible solution at low light intensities that do not supple enough energy and reductant for biomass formation.

**Figure 4:**
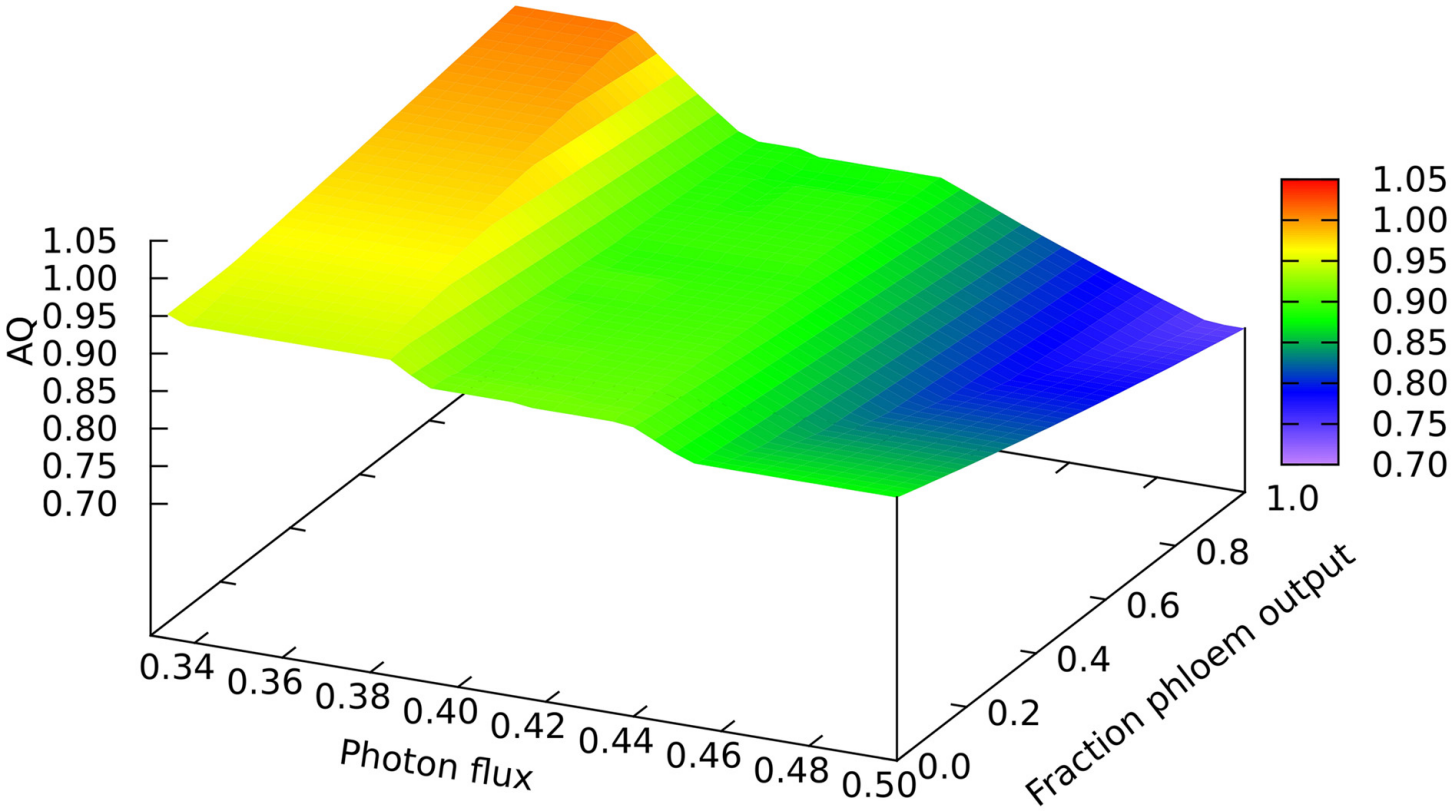
**Responses of the Assimilation Quotient to leaf output and light intensity**. The photon flux axis is light intensity from its minimal necessary value where only NH_3_ is used as N-source to a value just above the transition from region A into B.

In looking in detail at the changes in chloroplast and mitochondrial metabolism, the main qualitative difference appears to be in region B of the mitochondrial metabolism, where ATP production via Complex V, from reductant generated by malate dehydrogenase using imported malate, declines more rapidly with increase in light intensity when phloem sap is produced compared with biomass. This can be seen in the relative differences for the malate dehydrogenase flux (Figure 2) which range approximately 15% above and below the reference value, with the flux for biomass output generally higher (front edge of the plot) and that for phloem output lower (back edge). This figure also shows (at a photon flux of about 0.4 and a fractional phloem production of 0.6) that there can also be abrupt transitions in the solutions for the optimal flux distribution as the proportion of phloem output increases at the expense of biomass. Nevertheless, in general in Figures 2–**4**, the reaction fluxes change smoothly in the transition from 0 to 1 fractional output of phloem at any particular light intensity.

A clear quantitative difference is seen in the nitrogen uptake flux, which is larger for phloem sap production on account of the higher proportion of nitrogen than in biomass, as can be seen at the low end of the light range scanned in Figure 3 where all nitrogen is derived from ammonia. The lower demand for energy and reductant for phloem sap production from CO_2_ and NH_3_ means that it can be produced at slightly lower light levels, and that the transition to full NO_3_ utilization begins sooner for phloem than for biomass production (c.f. the front and back edges of the plot). However, the transition extends over a greater range of light flux for phloem production, reflecting the larger amount of reductant that has to be made in order to use 100% NO_3_. It is probably this increased demand for reductant that contributes to the more abrupt drop in mitochondrial malate dehydrogenase flux mentioned above.

Another quantitative difference between the growing leaf and the source leaf is in the values of the Assimilatory Quotient (AQ, CO_2_ fixed per O_2_ released). For the source leaf, AQ is 1.02 at minimal light where NH_3_ is the sole nitrogen source used, but falls to 0.74 at higher light levels where NO_3_ is being used instead. The corresponding figures for the expanding leaf are 0.97 and 0.88, as observed previously (Poolman et al., 2013). This illustrates how AQ responds to the difference between the composition of the nutrients and the metabolic outputs, and this dual dependence is illustrated in Figure 4 where AQ is plotted as a function of the transition in output from growth to phloem sap production and of light intensity in the range from minimally sufficient light to beyond the point where the transition from NH_3_ to NO_3_ use occurs. It is interesting that the nitrogen source has a bigger impact on the AQ for a source leaf than for an expanding leaf, presumably a reflection of differences in redox state and nitrogen content of the outputs. We should reiterate that in neither case is there any further change in AQ as light intensity and photorespiration increases (notably in regions C and D of Figure 1, upper panel—indicated by the phosphoglycolate export rate from the chloroplast). This implies, that at least for the optimal solutions in the range we have examined, that reassimilation of the CO_2_ released by photorespiration is linked to photosynthetic production of sufficient O_2_ to compensate for that used in the photorespiratory cycle.

## 4. Discussion

Our results have shown that the occurrence of transitions in the flux solutions for our rice leaf cell metabolism model at different light intensities are not peculiar to the production of a specific metabolic output, but are relatively insensitive to the nature of the output. Differences in the redox and energy demands for making different outputs do, however, cause small quantitative changes in the points at which the transitions occur.

Some of the quantitative differences in flux between production of biomass and production of phloem constituents arise because there is a small difference in the minimum amount of light needed to fix CO_2_ to give the required proportions of metabolites. Part of that difference comes from the known difference in redox state between the two mixtures, reflected in the Assimilatory Quotient values. Another component is in the amount of ATP required for synthesis, and we do not know this value with any certainty. In both cases, there will be a non-growth associated maintenance energy value, which might be assumed to be more or less constant. However, in the case of biomass formation, there is the ATP cost of polymerization that is not fully accounted for explicitly in our model, whereas in the case of phloem sap production, there is the ATP cost of export of sucrose and amino acids, which again is not explicitly represented. Given the uncertainties in estimating both of these values, we have effectively assumed they are equivalent, so any differences in ATP requirement reflect those of biosynthesis of the required mixes of small metabolites. In any case, the small difference in the minimum light input required in the two cases is of no significance compared to the light needed to satisfy the cell's maintenance ATP needs, which itself is a very uncertain figure, though the value we have used results in a minimum quantum demand figure that is in the range of experimental measurements.

Our AQ values for an expanding leaf are comparable to those measured experimentally, as is the magnitude of the difference between using NH_3_ and NO_3_ as N source (Searles and Bloom, 2003). The values for the mature leaf producing phloem sap are in the range of experimental measurements, but perhaps show a larger effect of the N source related to the higher ratio of N:C in the phloem output. As in our previous paper (Poolman et al., 2013), the optimal solutions we obtain show no change in AQ as the rate of photorespiration increases. As stated above, this implies that if photorespiratory CO_2_ is recovered, O_2_ consumption, and evolution are balanced for the complete cycle. Though we cannot claim on the basis of this evidence that this will always be the case in all feasible metabolic states, nevertheless the fact that there are situations where it can occur undermines the use of AQ as an indicator of the degree of photorespiration (e.g., Skillman, 2008). This issue merits further investigation.

However, in spite of the differences we have highlighted between the central carbon metabolism of the leaf when it is exporting phloem sap as compared to growing, the most striking finding is how little most of the fluxes in central carbon metabolism change in order to redirect the output. The flux patterns predicted by FBA for growing microorganisms do not appear to be highly sensitive to the precise biomass composition used as the output constraint. This is likely to be a significant component of the robustness in flux patterns that we see here, though it is possible that the compartmentation, especially of the chloroplast metabolism, further insulates the central metabolism from its eventual outputs. In terms of exploring the properties of plant metabolism models with FBA, this is an advantage to the modeler in that, since it is experimentally difficult to measure accurately the inputs and outputs of specific plant tissues, the relative insensitivity of the fluxes to this data compared with the sensitivity to light intensity gives some confidence that it is feasible to discern some general trends in the metabolic responses. It must be admitted nevertheless that the model results may over-state the degree of robustness, since all the solutions are obtained with the same optimization criterion which therefore selects particular regions of feasible metabolic space. Since the regulation of plant metabolism will have evolved to balance multiple optimization objectives, and may never perfectly satisfy any of them, then real plant cells may well show greater variability in their actual responses. Even so, we believe that modeling has a role to play in formulating detailed and coherent hypotheses about how plant metabolism could potentially behave, for comparison with experimental evidence (c.f. Williams et al., 2010; Cheung et al., 2013).

## Author contributions

The work was designed and conceived by David A. Fell, Mark G. Poolman, and Sudip Kundu. Mark G. Poolman developed ScrumPy and the Python modules used for the analyses reported here. All authors participated in developing and curating the rice metabolic model and running the analyses. The manuscript was drafted by David A. Fell and Mark G. Poolman and checked and approved by all authors.

### Conflict of interest statement

The authors declare that the research was conducted in the absence of any commercial or financial relationships that could be construed as a potential conflict of interest.
