# Supplementary material for: Metabolic trade-offs between biomass synthesis and photosynthate export at different light intensities in a genome–scale metabolic model of rice
Source: Front Plant Sci. 2014 Nov 28;5:656. doi: 10.3389/fpls.2014.00656 (PMC4246663; doi:10.3389/fpls.2014.00656)
Supplement: Supplementary file 1 [file DataSheet1.PDF]

Supplementary Material: Metabolic Modelling of RiceMetabolic Trade–offs between Biomass Synthesis and Photosynthate Export at Different Light Intensities in a Genome–Scale Metabolic Model of Rice

Mark G. Poolman [1], **Sudip Kundu** [2], **Rahul Shaw**[2] **and David A. Fell** [1,*]

[1] *Cell Systems Modelling Group, Department of Biological and Medical Science, Oxford Brookes University, Oxford, UK*
[2] *Department of Biophysics, Molecular Biology and Bioinformatics, Calcutta University, Kolkata, India*

Correspondence*:
David A. Fell
Department of Biological and Medical Science, Oxford Brookes University, Oxford, OX3 0BP, UK, dfell@brookes.ac.uk

1 SUPPLEMENTARY TABLES

Supplementary Table 1. Relative biomass fluxes for production of leaf material. Starch flux calculated in terms of GLC monomeric units. Conol, Coumol and Sinap are CONIFERYL-ALCOHOL, COUMARYL-ALCOHOL" and SINAPYL-ALCOHOL respectively, other metabolites are their BioCyc identifiers.

Metabolite	Flux (Mol.gdw^{-1}/unit time)
ALA	6.83×10^{-05}
AMP	3.60×10^{-06}
ARG	8.36×10^{-06}
ASN	3.60×10^{-05}
ASP	3.57×10^{-05}
CEL	3.05×10^{-03}
CMP	3.87×10^{-06}
CYS	7.90×10^{-06}
Conol	9.24×10^{-05}
Coumol	1.11×10^{-04}
GLC	1.39×10^{-05}
GLN	3.83×10^{-05}
GLT	3.81×10^{-05}
GLY	2.12×10^{-05}
GMP	3.44×10^{-06}
HIS	5.45×10^{-06}
ILE	1.34×10^{-05}
LEU	2.63×10^{-05}
LINO	3.57×10^{-05}
LYS	5.69×10^{-06}
MET	1.47×10^{-06}
PHE	9.99×10^{-06}
PRO	4.34×10^{-06}
SER	4.04×10^{-05}
STARCH	1.11×10^{-04}
SUC	2.19×10^{-05}
Sinap	7.92×10^{-05}
THR	2.88×10^{-05}
TMP	3.88×10^{-06}
TRP	1.08×10^{-06}
TYR	8.58×10^{-06}
UMP	3.86×10^{-06}
VAL	8.87×10^{-06}
dAMP	3.77×10^{-06}
dCMP	4.07×10^{-06}
dGMP	3.60×10^{-06}

Supplementary Table 2. Relative 'biomass' fluxes under production of phloem. An additional flux to starch was set such that total C flux to starch was equal to total C flux to phloem, so that phloem production can be supported at night.

Metabolite	Flux (Mol.gdw^{-1}/unit time)
ALA	1.83×10^{-05}
ARG	9.58×10^{-05}
ASN	1.72×10^{-04}
ASP	1.21×10^{-04}
GLN	1.89×10^{-04}
GLT	1.56×10^{-04}
HIS	9.69×10^{-06}
ILE	2.04×10^{-05}
LEU	1.72×10^{-05}
LYS	5.16×10^{-05}
PHE	4.30×10^{-06}
PRO	1.29×10^{-05}
SER	1.16×10^{-04}
STARCH	2.07×10^{-03}
SUC	6.18×10^{-04}
THR	4.40×10^{-05}
TYR	5.39×10^{-06}
VAL	4.32×10^{-05}
